# Cardiac Conduction Defects Induced by Paracetamol Overdose: A Rare Case

**DOI:** 10.7759/cureus.89877

**Published:** 2025-08-12

**Authors:** Aastha Arunkumar Patel, Priyank Shah, Mildred Chikaodinaka Odukwu, Kirtan Soni, Dhruvil Vinaybhai Patel, Venkata Sai Harshabhargav Chenna, Prachi Dawer, Ewuradjoa Ayirebi-Acquah

**Affiliations:** 1 Internal Medicine, Gujarat Medical Education &amp; Research Society (GMERS) Medical College, Gandhinagar, Gandhinagar, IND; 2 Internal Medicine, Shiv Hospital, Patdi, IND; 3 Internal Medicine, Afe Babalola University, Ado-Ekiti, Ado-Ekiti, NGA; 4 Medicine, University of Perpetual Help System DALTA, Las Pinas, PHL; 5 Internal Medicine, University College of Medical Sciences, New Delhi, IND; 6 Internal Medicine, Lekma Hospital, Accra, GHA

**Keywords:** acute liver failure, cardiac complications, n-acetylcysteine, paracetamol overdose, second-degree av block

## Abstract

Paracetamol, also known as acetaminophen, is one of the most frequently used analgesic and antipyretic agents worldwide and is considered safe at therapeutic doses. However, in overdose situations, it is a leading cause of acute liver failure. While hepatic and renal toxicities are well documented, cardiac complications are rarely reported. We present a rare case of a 32-year-old woman who developed transient second-degree atrioventricular (AV) block following intentional ingestion of 15 grams of paracetamol. Although her liver function tests were markedly elevated and she presented with classical signs of hepatotoxicity, she also experienced bradycardia and intermittent AV block during hospitalization. With timely administration of N-acetylcysteine and supportive management, including correction of electrolyte imbalances, the conduction defects resolved without further complications. This case highlights the need to consider cardiac monitoring in patients with significant paracetamol overdose, especially those with evidence of liver dysfunction.

## Introduction

Paracetamol (PCM), commonly referred to as acetaminophen, is widely regarded as a safe over-the-counter analgesic and antipyretic when used within recommended dosing limits. Despite its favorable safety profile, PCM overdose remains one of the leading causes of acute liver failure worldwide, particularly in cases of intentional self-poisoning [1]. The toxic threshold for hepatic injury is generally considered above 7.5-10 g in adults within 24 hours (approximately >150 mg/kg body weight) and above 200 mg/kg in children as a single dose [2].

While hepatotoxicity is the predominant clinical concern following overdose, cardiovascular complications such as conduction abnormalities, arrhythmias, and myocarditis have been sporadically reported, predominantly in the context of massive ingestions often exceeding 25 g [1,3,4]. Such complications are exceedingly rare, with the exact incidence unknown due to limited large-scale epidemiological data. Available literature, largely comprising case reports and small case series, suggests that cardiac manifestations may occur in less than 1% of PCM overdose cases but may be under-recognized due to overshadowing hepatic pathology and lack of routine cardiac monitoring [5-7].

The pathophysiology of PCM-induced cardiotoxicity remains incompletely understood but may involve direct myocardial toxicity from toxic metabolites, systemic inflammatory responses, metabolic disturbances, and electrolyte imbalances secondary to liver failure [5,7]. Notably, conduction defects such as transient atrioventricular (AV) blocks have been documented but remain rare clinical entities. This report describes a case of transient second-degree AV block following ingestion of 15 grams of PCM, highlighting the need for vigilance for cardiac complications even at doses lower than those traditionally associated with severe toxicity.

## Case presentation

A 32-year-old female, weighing 54 kg, with no prior medical or psychiatric history, presented to the emergency department following an intentional ingestion of 15 grams of PCM (approximately 278 mg/kg). She was brought in approximately 6 hours post-ingestion and reported symptoms of nausea, vomiting, mild upper abdominal pain, and confusion. She denied any co-ingestion of other substances or prior cardiovascular symptoms. On initial examination, she was hemodynamically stable but had a pulse rate of 54 beats per minute. Her oxygen saturation and temperature were within normal limits.

**Figure 1:**
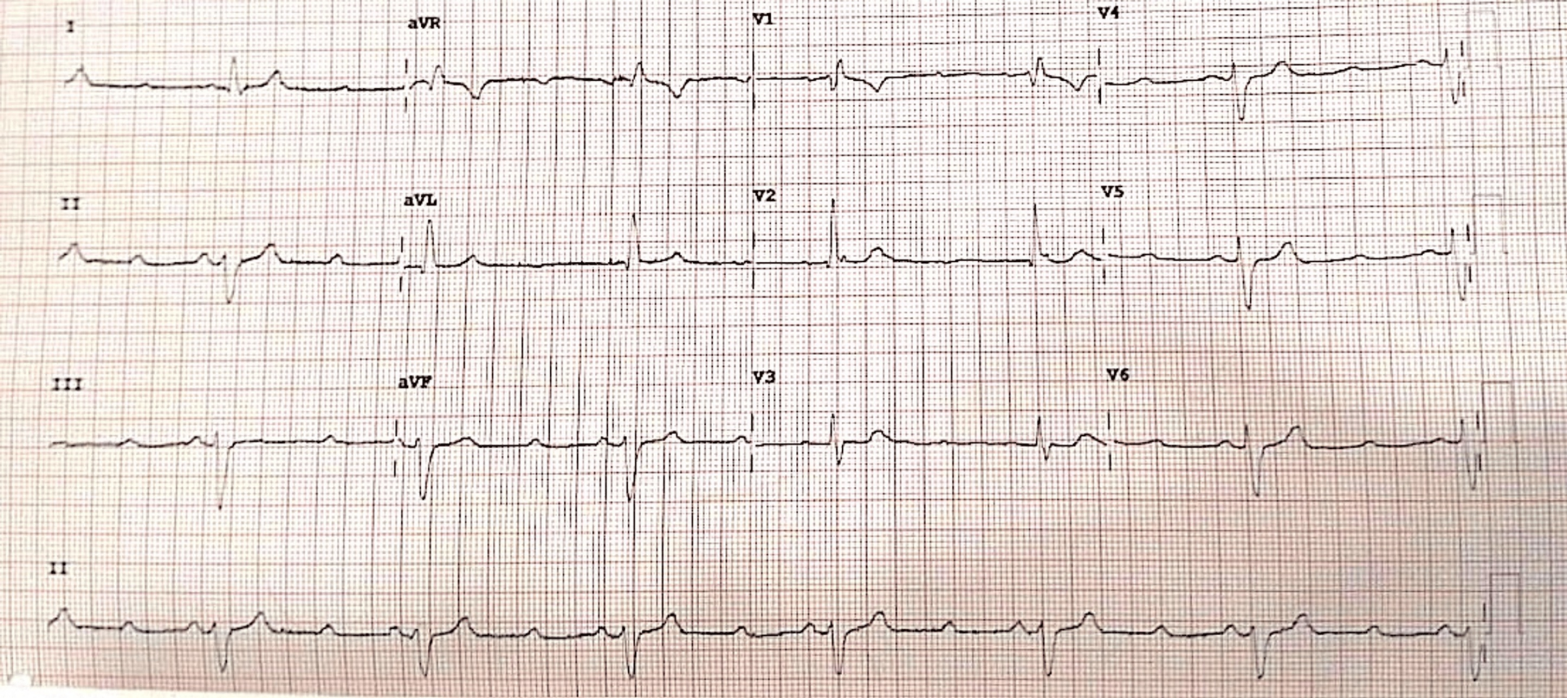
A 12-lead ECG showing Mobitz type I second-degree atrioventricular block in a 32-year-old female after paracetamol overdose, resolving with supportive care and electrolyte correction.

Laboratory workup revealed significantly elevated liver enzymes with aspartate transaminase (AST) at 945 U/L and alanine transaminase (ALT) at 1,120 U/L, along with a mildly elevated international normalized ratio (INR) of 1.7. Her serum PCM level was 290 μg/mL, well above the toxic threshold. Electrolyte analysis demonstrated mild hypokalemia (K⁺: 3.1 mmol/L) and hypomagnesemia (Mg²⁺: 1.5 mg/dL). She was immediately initiated on intravenous N-acetylcysteine (NAC) therapy. On the second day of hospitalization, the patient began exhibiting episodes of lightheadedness and bradycardia. Telemetry revealed intermittent second-degree AV block (Mobitz type I), which was confirmed on a 12-lead ECG (Figure 1). Cardiac troponins were within normal limits, and an echocardiogram showed normal cardiac structure and function.

No specific anti-arrhythmic therapy was initiated. Instead, her electrolyte imbalances were corrected - potassium increased from 3.1 mmol/L to 4.2 mmol/L and magnesium from 1.5 mg/dL to 2.0 mg/dL over 24 hours - and close cardiac monitoring continued. Over the following 48 hours, the AV block resolved spontaneously, and her heart rate normalized. Her liver function tests began trending downwards, and she was discharged on day 7 in stable condition after psychiatric consultation.

## Discussion

The hepatotoxic effects of PCM overdose are well-characterized and primarily result from the metabolic pathway involving cytochrome P450 enzymes, which convert a small fraction of PCM to the highly reactive compound NAPQI. This intermediate is typically neutralized by conjugation with glutathione. In overdose situations, however, the body’s glutathione reserves are depleted, allowing NAPQI to accumulate and cause cellular injury through oxidative stress and mitochondrial dysfunction [5,8].

Although hepatic toxicity is the most prominent concern in PCM overdose, there is emerging recognition of its potential to cause extrahepatic complications, including cardiac toxicity. Reports in the literature have documented arrhythmias such as ventricular tachycardia, bradycardia, QT interval prolongation, and varying degrees of AV block in the setting of PCM poisoning. In a case-based review, KhabazianZadeh et al. described incidents of myocardial injury and conduction defects associated with PCM toxicity, emphasizing their rarity and reversibility [1]. Similarly, Ralapanawa et al. examined PCM cardiotoxicity and concluded that the data, although limited, support a potential link between PCM overdose and cardiac conduction abnormalities [7]. Several published cases have documented cardiotoxic effects associated with PCM overdose, including myocardial dysfunction, various arrhythmias, and conduction abnormalities such as AV block, as summarized in Table 1.

**Table 1:** Summary of published cases and studies describing cardiotoxicity associated with paracetamol (acetaminophen) overdose, including clinical manifestations, proposed mechanisms, and clinical implications.

Reference	Summary of Cardiotoxicity Cases	Mechanism Proposed	Clinical Relevance
KhabazianZadeh et al., 2019 [1]	Case-based review reporting myocardial dysfunction, conduction abnormalities, and cardiogenic shock following acetaminophen overdose	Direct myocardial toxicity, oxidative stress, inflammation	Highlights rare but severe cardiac complications requiring monitoring.
Senaratne et al., 2021 [6]	Review on ventricular arrhythmias in critical care, mentioning paracetamol-induced arrhythmias as a potential etiology	Oxidative stress and membrane disruption	Importance of electrolyte correction and close monitoring highlighted
Ralapanawa et al., 2016 [7]	Observational study showing ECG changes and biochemical markers of myocardial injury in paracetamol poisoning patients	Myocardial injury secondary to metabolic disturbances	Emphasizes the need for cardiac evaluation in severe overdose cases
Craig et al., 2011 [8]	Study on overdose pattern and outcomes, including rare cardiac complications with acute liver failure from paracetamol toxicity	Systemic inflammatory response and mitochondrial dysfunction	Supportive care including cardiac monitoring recommended

The pathophysiology behind PCM-induced cardiac conduction abnormalities is likely multifactorial. Hepatic injury often leads to metabolic and electrolyte disturbances, especially hypokalemia, hypomagnesemia, and hypocalcemia, which are known to destabilize myocardial membrane potentials and predispose to arrhythmias [9,10]. Additionally, systemic inflammation resulting from liver injury may impair cardiac excitability [11]. There is also a possibility of direct cardiotoxicity.

In this case, the patient was administered intravenous NAC according to the standard 21-hour protocol, starting with a loading dose of 150 mg/kg over 1 hour, followed by 50 mg/kg over 4 hours, and then 100 mg/kg over the remaining 16 hours. Cardiac monitoring was initiated immediately upon admission, utilizing continuous telemetry throughout hospitalization. A 12-lead ECG was performed on admission and repeated daily during the period of elevated liver enzymes and conduction abnormalities until resolution of the AV block. Electrolytes were closely monitored every 12 hours and corrected as needed to maintain potassium and magnesium levels within normal ranges.

The patient’s presentation of second-degree AV block and bradycardia coincided with peak hepatic enzyme elevation and metabolic derangements. The resolution of conduction abnormalities following electrolyte correction and NAC therapy supports a reversible, non-structural etiology. Although invasive interventions such as pacemaker insertion were not required, the favorable outcome may have depended on vigilant cardiac monitoring. This underscores the importance of continuous telemetry in all patients with moderate-to-severe PCM toxicity, especially those demonstrating hepatic dysfunction [12-16]. Animal studies indicate that NAPQI may disrupt calcium homeostasis and mitochondrial respiration in cardiomyocytes, leading to decreased contractility and altered conduction [15].

Furthermore, insights from other liver failure models, such as acute liver failure from viral hepatitis or drug-induced liver injury, reveal similar electrophysiological manifestations. These include prolonged QT intervals, bradyarrhythmias, and even sudden cardiac death, often linked to cytokine storms, oxidative stress, and impaired autonomic regulation [13,14]. Such parallels suggest that PCM-induced hepatic injury may follow similar downstream cardiac effects.

In addition to the clinical and pathophysiological insights, incorporating the patient’s perspective highlights the importance of comprehensive care beyond the biomedical model. Our patient expressed significant anxiety regarding both the overdose and the cardiac complications, underscoring the psychological impact of PCM poisoning. She appreciated clear communication about her condition and the rationale for continuous cardiac monitoring and electrolyte correction. The multidisciplinary approach, including mental health support post-discharge, was vital in addressing both her physical recovery and psychological well-being. This patient-centered dimension reinforces that clinicians should consider the emotional and psychosocial needs of patients when managing complex overdose cases, promoting better adherence and holistic recovery.

Given the increasing incidence of PCM overdose, particularly in low-resource settings where it is easily accessible, awareness of such rare complications is vital. A multidisciplinary approach involving toxicologists, hepatologists, and cardiologists can enhance patient outcomes. Additionally, clinicians should advocate for early ECG evaluation and telemetry in any PCM overdose case that demonstrates biochemical signs of hepatic involvement, regardless of whether the patient reports cardiac symptoms.

Limitations

This case report describes a young, otherwise healthy female patient with PCM overdose resulting in transient cardiac conduction defects. The findings may have limited generalizability to broader populations, particularly older patients or those with pre-existing cardiovascular or systemic comorbidities, which could influence the severity and presentation of cardiac complications. Additionally, the dose ingested in this case (15 grams) is significant but lower than some previously reported massive overdoses associated with cardiotoxicity; thus, outcomes might differ with higher doses or delayed treatment. Larger prospective studies are needed to better understand the incidence, risk factors, and clinical course of PCM-induced cardiac effects across diverse patient groups.

## Conclusions

While PCM is widely regarded as a safe analgesic and antipyretic, its toxicity extends beyond hepatic injury in overdose, with emerging evidence of transient cardiac conduction disturbances such as AV block. The underlying mechanisms likely involve a combination of toxic metabolites, metabolic derangements, and possible direct myocardial effects. Clinicians managing significant PCM overdoses should maintain a high index of suspicion for cardiac complications and implement continuous cardiac telemetry monitoring for at least 72 hours post-ingestion. Serial 12-lead ECGs are recommended on admission and at 12- to 24-hour intervals or sooner if clinical status changes. Prompt correction of electrolyte imbalances and timely administration of NAC remain critical components of care. Increased awareness and further research are essential to better understand the incidence and pathophysiology of PCM-induced cardiac effects, which may currently be underdiagnosed.
